# Relationship between oral health-related knowledge, attitudes, practice, self-rated oral health and oral health-related quality of life among Chinese college students: a structural equation modeling approach

**DOI:** 10.1186/s12903-021-01419-0

**Published:** 2021-03-06

**Authors:** Suge Zheng, Lili Zhao, Nianting Ju, Tiantian Hua, Shunhua Zhang, Shengkai Liao

**Affiliations:** 1 School of Medical Imaging, Bengbu Medical College, Bengbu, 233030 Anhui China; 2 Department of Stomatology, First Affiliated Hospital of Bengbu Medical College, Bengbu, 233004 Anhui China

**Keywords:** Knowledge-attitudes-practice, Self-rated oral health, Oral health-related quality of life, Structural equation modeling, Chinese college students

## Abstract

**Background:**

This study aimed to evaluate the associations among oral health-related knowledge, attitudes, practice (KAP), self-rated oral health and oral health-related quality of life (OHRQoL) among Chinese college students.

**Methods:**

Of the 2000 participants, 1751 (87.55%) students answered an online questionnaire between October 2019 and January 2020. The questionnaire included demographic characteristics, knowledge, attitudes, and practice related to oral health, self-rated oral health, and OHRQoL. Structural equation modelling was applied to assess the associations among study variables.

**Results:**

Among the total students, oral health-related knowledge and attitudes were satisfactory, while the oral health practice was not optimistic. The final model showed satisfactory fitness to the data. Oral health knowledge was associated with attitudes directly and positively. Attitudes toward oral health had a direct and positive effect on practice. Oral health knowledge had an indirect effect on practice through attitudes. Oral health practice was directly associated with self-rated oral health. Oral health knowledge, practice, and self-rated oral health all affected OHRQoL directly and positively, while attitudes had a direct negative impact on OHRQoL.

**Conclusions:**

OHRQoL was influenced by oral health knowledge, attitudes, practice, and self-rated oral health. Our findings support the KAP theory. Limitations of the KAP model were also found.

## Background

Good oral health is an essential component to maintain and improve general health and quality of life. Oral health-related quality of life (OHRQoL) can be used to measure the impact of oral health on an individual’s quality of life [1–3]. OHRQoL represents the subjective perception of oral conditions and subjective evaluation of physical function, psychological function, and social activity aspects of oral health [4–6]. OHRQoL can also assess the relationship between oral status and general health from subjective perspectives and experiences [7–9].

It is important to have a good OHRQoL for college students, who play a significant role in the future development of a country, eventually becoming the future promoters of social progress [10, 11]. Compared with middle-aged people, young college students are in a dynamic growth period, and their health, social psychology, lifestyles, and behaviours are more likely to change [11, 12]. Poor OHRQoL can negatively affect college students' oral health condition and general well-being in the future [13, 14]. Therefore, it is particularly important to identify factors associated with the OHRQoL of college students, in which it is conducive to determine oral health promotion intervention strategies [15].

Assessment of self-rated oral health is considered a valid and useful measurement indicator of oral health conditions in epidemiology, which can easily and simply evaluate the individual general oral health status [16–18]. Information on self-rated oral health can help dental clinicians determine routine diagnostic procedures [19] and assess the demand for treatment [20]. In addition, the evaluation of self-rated oral health can help individuals recognise the importance of regular dental check-ups [21].

Self-rated oral health is related to subjective perception factors and clinical oral factors [19–21]. Subjective perception factors include reported general oral health status and the presence of abnormal oral symptoms [20]. They are thought to affect self-rated oral health, which can further influence participants' quality of life [11, 19]. In addition, a study done by Yamane, et al. in Japan identified that self-related oral health was associated with OHRQoL in Japanese young adults [18]. However, few studies have been conducted to demonstrate the relationship between OHRQoL and self-rated oral health among college students in China.

Knowledge, attitudes, and practices (KAP) theory is one of the theoretical models used to change human health-related behaviours [22]. The KAP theory holds that healthy knowledge is the basis for establishing positive attitudes and healthy behaviours, and attitudes are the driving force of behavioural change, and promoting healthy behaviours is the goal [22]. Previous studies assessed the status of oral health-related knowledge, attitudes, and practices targeting different groups of people [23–26]. However, few studies have been conducted on the strength of the relationships between knowledge, attitudes, and practice related to oral health [18].

According to KAP theory, there is a causal relationship between knowledge, attitudes, and practice [27]. However, knowledge, attitudes, and practice are all potential variables that are difficult to measure directly. The traditional multivariate statistical method can only identify the factors that affect knowledge, attitudes, and practice, but cannot elucidate the complex causal relationships involved in the process. In comparison, structural equation modelling (SEM) is a far superior statistical technique, which can compensate for the defects of traditional statistical methods. This multivariate statistical analysis technique can deal with potential variables, observation indexes, and measurement errors simultaneously [28–30]. Furthermore, it can explore the causal relationships among potential variables and quantitatively evaluate the direct and indirect effects of variables [28–30]. To date, the SEM approach has been widely used in psychology, behavioural and social science, biomedicine, management, and other fields [31, 32].

This study aimed to evaluate the associations among oral health-related knowledge, attitudes, practice, self-rated oral health, and OHRQoL in a group of Chinese college students based on the KAP theory using an SEM approach.

## Methods

### Subjects and design

We used the convenience sampling technique to conduct this cross-sectional survey.

The sample size was calculated using the population proportion statistical formula [33]. *N* = *Z*^2^*P* (1 − *P*) *d*^2^ (*Z* = 1.96, *P* = 60.1%, *d* = 0.05), where *Z* = critical value corresponding to 95% confidence level = 1.96; *P* = Proportion with parameter (the awareness rate of oral health knowledge = 60.1%, which was from a previous study [34]). Therefore, the calculated sample size was 368 and, after considering a 20% non-response rate, the minimum required sample size was 441. However, a total sample size of 1751 was used for this study. Our sample size exceeded the minimum requirement. A sufficient sample size ensures the credibility of the research results.

College students were invited to participate in this study from a total of 22 universities throughout the northern (8 universities), southern (7 universities), and central (7 universities) regions of the Anhui Province located in the east of China. Participating universities were those that had responded positively to invitations to help collect data. Data were collected from 1 October 2019 through 26 January 2020.

We conducted a web-based questionnaire. The link to the online questionnaire was sent to administrators from the invited universities for distribution to registered college students. The link to the survey was also sent to students’ social media groups or forums. Furthermore, we encouraged the students who had received an online questionnaire to forward the link to their classmates. After providing informed consent, participants completed the anonymous online questionnaire independently. On average, questionnaires were completed within approximately 10–15 min.

The inclusion criteria were as follows: (1) no reading and comprehension disability; and (2) voluntary participation in the study and providing oral informed consent. The exclusion criteria were as follows: (1) incomplete data; (2) mental illnesses; and (3) reluctance to participate after the study was explained.

In total, 2000 college students were recruited for this study. A total of 1817 students completed the online questionnaire, with a response rate of 90.85%. However, 66 questionnaires were excluded because of too many missing values (> 10%). Ultimately, a total of 1751 complete and valid questionnaires were collected with an effective response rate of 87.55%.

### Survey instruments

The original questionnaire was developed referring to the relevant literature review and the 4th National Oral Health Survey in the Mainland of China [34]. The items in the questionnaire were validated by several experts. The questionnaire was pre-tested on randomly sampled college students before the formal survey. The questionnaire was improved according to the results of the preliminary investigation and opinions of experts.

The questionnaires addressed socio-demographic characteristics, oral health-related knowledge, attitudes, practice, self-rated oral health, and OHRQoL.

The question ‘In general, how would you evaluate your oral health?’ was used to assess self-rated oral health. It was measured using a Likert scale with five options, whereby 1 was ‘very good’, and 5 was ‘very poor’ [11, 18]. Low scores reflected a good self-assessment of oral condition.

Oral health-rated knowledge consists of 9 items, for example ‘Plaque can cause tooth decay and periodontal disease, including gingivitis and periodontitis’ (K1); ‘Fluoride toothpaste can prevent caries’ (K2); ‘See your dentist or hygienist for regular dental exams that can help detect oral problems early and maintain your dental health’ (K3); ‘A regular dental deep cleaning using the ultrasonic dental scaler can maintain good oral hygiene’ (K4); ‘In the early stages of gingivitis, the gums bleed when brushing or biting hard objects’ (K5); ‘Acute pulpitis can produce intense spontaneous pain, nocturnal pain’ (K6); ‘Caries are often characterized by irritation, biting discomfort or pain’ (K7); ‘Halitosis is mainly caused by oral diseases’ (K8) and ‘Pit and fissure sealants can protect the teeth and prevent dental caries’ (K9). Response options were ‘True’ or ‘False’. The correct answer was scored 1, and incorrect answers were scored 0. The final scores of oral health-rated knowledge ranged from 0 to 9. Higher scores indicated better oral health knowledge. The total awareness rate of oral health knowledge was equal to the total number of knowledge questions answered correctly/(the number of knowledge items in each questionnaire × the number of effective response participants) × 100%. The awareness rate of each oral health knowledge question was equal to the number of participants answered correctly/the number of effective response participants × 100%.

Attitudes toward oral health care were measured using 4 items. They included ‘Do you think that oral diseases can harm the general health?’ (A1); ‘Do you think eating sweets (such as cakes, biscuits and juices) and carbonated drinks will not cause tooth decay?’ (A2); ‘Do you think regular cleaning is good for oral health?’ (A3) and ‘Do you think oral diseases can be prevented?’ (A4). The possible response was three options, whereby 0 was ‘Disagree’, 1 was ‘Uncertain’, and 2 was ‘Agree’. The final scores of attitudes toward oral health ranged from 0 to 8. Higher scores represented more positive attitudes toward oral health. The total holding rate of positive attitudes toward oral health was equal to the total number of positive attitudes questions/(the number of attitudes items in each questionnaire × the number of effective response participants × 100%. The holding rate of each positive attitudes questions was equal to the number of participants who opted ‘Agree’/the number of effective response participants × 100%.

Oral health practice was assessed using 8 items, which included ‘Do you brush your teeth more than twice a day?’ (P1); ‘Is your brushing time ≥ 3 min each time?’ (P2); ‘Do you often use fluoride toothpaste?’ (P3); ‘Do you think regular oral check-ups are good for oral health?’ (P4); ‘Do you change a toothbrush every three months?’ (P5); ‘Do you often use dental floss (or an interdental brush) to help clean your teeth?’ (P6); ‘Is your brushing method recommended by the Chinese association of stomatology as “horizontal vibrating brush method”?’ (P7) and ‘Do you often gargle after meals?’ (P8). Dichotomic answers: ‘Yes’ or ‘No’. Each of the items was scored 1 if conducted by the participant. The final scores of oral health practice ranged from 0 to 8.

Higher scores indicated better oral hygiene practices. The total execution rate of right oral health practice was equal to the total number of practice questions opted ‘Yes’/(the number of practice items in each questionnaire × the number of effective response participants) × 100%. The execution rate of each right oral health practice was equal to the number of participants opted ‘Yes’/the number of effective response participants × 100%.

Confirmatory factor analysis (CFA) and Cronbach's alpha were used to confirm the validity and reliability of the questionnaire. Cronbach's α for the KAP was α = 0.737.

The OHRQoL was assessed using the Chinese version of the OHIP-14 in our study [35] (Table S2). It can capture functional and psychosocial impairment aggravated by an oral health condition [36]. OHIP was first designed by Slade in 1994 [37], which contains 49 entries, namely OHIP-49. Three years later, Slade developed a short version of OHIP-14 based on OHIP-49 [1], which had good reliability, validity, and accuracy [38]. The OHIP-14 includes 14 items that explore the following 7 conceptual dimensions: functional limitation, physical pain, psychological discomfort, physical disability, social disability, and perceived handicap to measure the self-reported frequency of discomfort symptoms [39]. The responses included ‘very often = 4’, ‘often = 3’, ‘sometimes = 2’, ‘rarely = 1’ or ‘never = 0’ according to a five-point Likert scale. The higher the score on the OHIP-14, the worse the oral health status [40–42]. In this study, Cronbach’s α for the OHIP-14 CHN was α = 0.97.

### Statistical analysis

IBM® SPSS® Statistics 25.0 and IBM® SPSS® Amos™ 24.0 were used for data analysis.

Mean ± standard deviation or frequency and percentage were used to describe the demographic information and oral health status of the participants.

We used the skewness–kurtosis test to check the normality of the study variables. The sample data showed a normal distribution, where the absolute value of skewness was < 3 and the absolute value of kurtosis was < 8 [43].

Spearman’s correlation was used to evaluate correlations between latent variables. All differences were assessed using two-tailed tests, and the significance level was set at *p* < 0.05.

A structural equation model was constructed to determine the relationship between oral health knowledge, attitudes, practice, self-rated oral health, and OHRQoL.

The bootstrap method [44] was used to test the significance of the mediating effect of related variables in the ideal model. In addition, a bias-corrected bootstrap 95% confidence interval (CI) was used to examine the significance of direct and indirect effects [45, 46].

The maximum likelihood estimate (MLE) was used for parameter estimation, and the test level was set to α = 0.05. We used the root mean square error of approximation (RMSEA), goodness of fit index (GFI), adjusted goodness of fit index (AGFI), comparative fit index (CFI), incremental fit index (IFI), normed fit index (NFI), chi-square/degrees of freedom (χ^2^/df), and other indicators to evaluate the fitting effect of the model [47, 48].

**Fig. 1:**
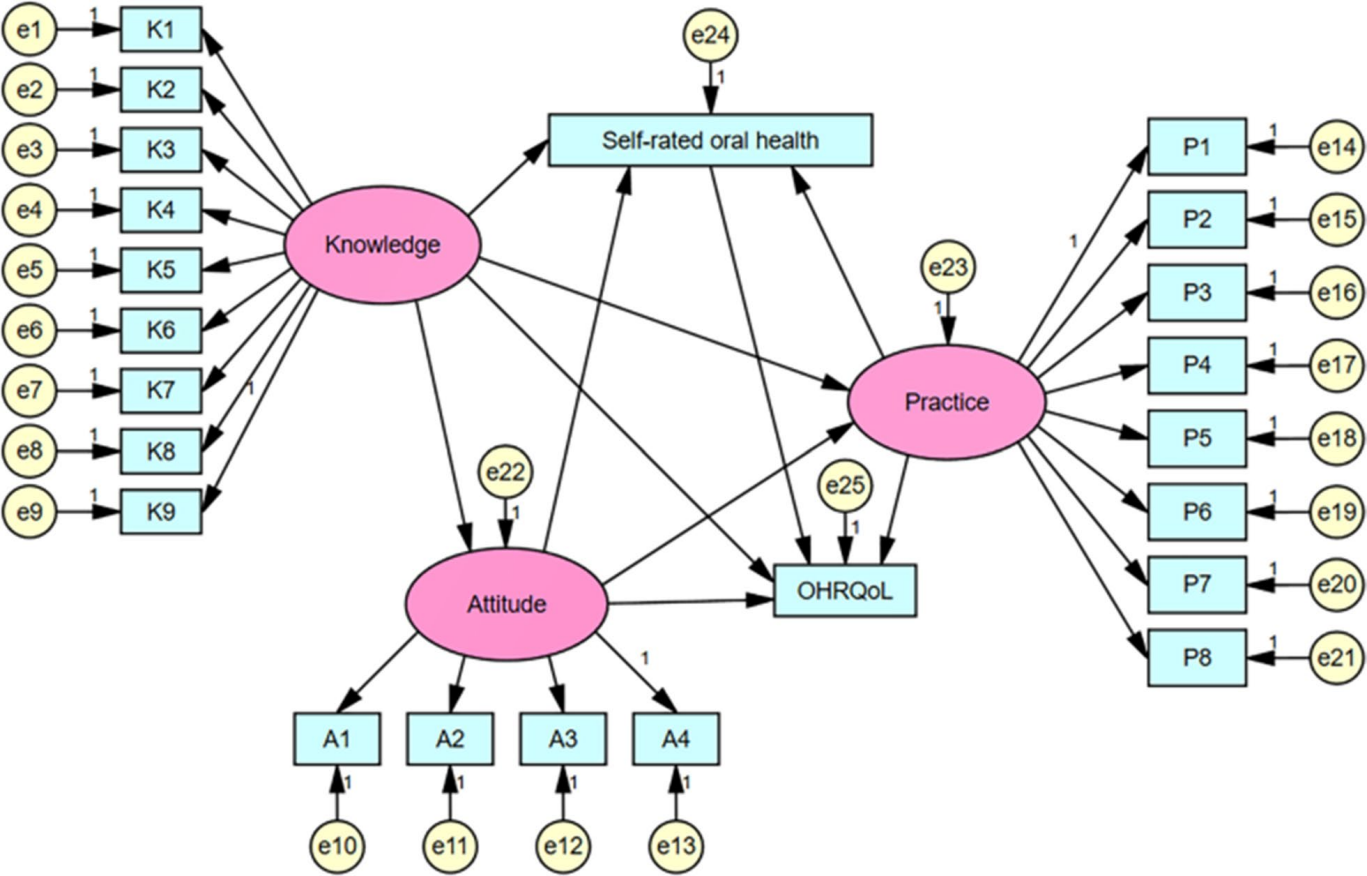
The ideal SEM. Rectangles show observed variables, ellipses indicate potential variables, and circles represent residual terms

According to our hypothesis, Fig. 1 shows the ideal SEM of association among oral health-rated knowledge, attitudes, practice, self-rated oral health, and OHRQoL in a sample of college students in China.

We removed the corresponding paths, because the path coefficients of ‘knowledge’ on ‘self-rated oral health’, ‘knowledge’ on ‘practice,’ and ‘attitudes’ on ‘self-rated oral health’ were not statistically significant in the ideal SEM fitting results (All *p* > 0.05).

In addition, the covariant relationships between e3 and e4 were established. Through repeated modification and fitting of the model, the fit indices of SEM finally reached the adaptation standards: χ^2^/*df* = 3.459, RMSEA = 0.037, GFI = 0.961, AGFI = 0.951, NFI = 0.900, IFI = 0.927, CFI = 0.926, PGFI = 0.780, PNFI = 0.797, and PCFI = 0.820.

The Cronbach's α value of the final SEM was 0.703 and the KMO value was 0.879, showing good reliability and validity [49, 50].

## Results

### Sample characteristics

As a result, we analysed data from 1751 college students (757 males, 994 females) aged 22.01 ± 4.83 years in this study. Overall, 17.76% of the participants had a healthier self-rated oral status, of which 4.97% were very good, and 12.79% were good. The mean total OHIP-14 score of the participants was 13.29 ± 12.15. Table 1 shows the specific demographic data, self-rated oral health, and mean total OHIP-14 score.

**Table 1:** Demographic characteristics, self-rated oral health and OHIP-14 score of participants (n = 1751)

Variable	Number	Constituent ratio/%
Gender		
Male	757	43.23
Female	994	56.77
Major		
Stomatology major	277	15.82
Medical non-oral specialty	651	37.18
Non-medical major	823	47.00
Annual household economic income		
< ¥50,000	755	43.12
¥50,000–120,000	717	40.95
> ¥120,000	279	15.93
Family origin		
Rural areas	996	56.88
Urban areas	755	43.12
Educational level		
Freshman	464	26.50
Sophomore	251	14.33
Junior	482	27.53
Senior	201	11.48
Graduate	172	9.82
First-year graduate	78	4.45
Second-year graduate	41	2.34
Third-year graduate	62	3.54
Only-child or not		
Yes	532	30.38
Two	1027	58.65
Three	136	7.77
Four or more	56	3.20
Self-rated oral health		
Very good	87	4.97
Good	224	12.79
Fair	1160	66.25
Poor	243	13.88
Very poor	37	2.11
Oral Health Impact Profile-14		
Total		13.29 ± 12.15^a^
Age		22.01 ± 4.83

^a^Mean ± SD

### Descriptive analysis for oral health-related knowledge, attitudes, practice

The total awareness rate of oral health knowledge among college students was 86.26%. The total holding rate of positive attitudes toward oral health was 78.36%, which was slightly lower than that of knowledge, but most participants had favourable attitudes. The total execution rate of right practice toward oral health was 45.43%. The specific values are shown in Table 2.

**Table 2:** Descriptive statistics for oral health-related knowledge, attitudes, practice

	M ± SD (range)	N (%)		M ± SD (range)	N (%)		M ± SD (range)	N (%)
K1	0.90 ± 0.30 (0–1)	1574 (89.89)	A1	1.71 ± 0.58 (0–2)	1362 (77.78)	P1	0.74 ± 0.44 (0–1)	1292 (73.79)
K2	0.79 ± 0.41 (0–1)	1381 (78.87)	A2	1.43 ± 0.85 (0–2)	1162 (66.36)	P2	0.57 ± 0.50 (0–1)	993 (56.71)
K3	0.88 ± 0.32 (0–1)	1543 (88.12)	A3	1.78 ± 0.56 (0–2)	1496 (85.44)	P3	0.42 ± 0.49 (0–1)	744 (42.49)
K4	0.85 ± 0.36 (0–1)	1489 (85.04)	A4	1.76 ± 0.59 (0–2)	1468 (83.84)	P4	0.78 ± 0.41 (0–1)	1367 (78.07)
K5	0.89 ± 0.31 (0–1)	1565 (89.38)				P5	0.20 ± 0.40 (0–1)	351 (20.05)
K6	0.90 ± 0.30 (0–1)	1573 (89.83)				P6	0.37 ± 0.48 (0–1)	652 (37.24)
K7	0.90 ± 0.31 (0–1)	1568 (89.55)				P7	0.39 ± 0.49 (0–1)	681 (38.89)
K8	0.83 ± 0.38 (0–1)	1452 (82.92)				P8	0.16 ± 0.37 (0–1)	284 (16.22)
K9	0.83 ± 0.38 (0–1)	1448 (82.70)						
K	7.76 ± 2.02 (0–9)	86.26%	A	6.68 ± 1.56 (0–8)	78.36%	P	3.63 ± 1.91 (0–8)	45.43%

M, Mean; SD, Standard Deviation; K, Total awareness rate of oral health knowledge; A, Total holding rate of positive attitudes toward oral health; P, Total execution rate of right practice toward oral health

### Correlation analysis among latent variables

There were positive correlations between oral health-related knowledge, attitudes, and practice (*r* = 0.437, 0.162, 0.095, all *p* < 0.01). The results are shown in Table 3.

**Table 3:** Correlation analysis among latent variables

	Knowledge	Attitudes	Practice
Knowledge	–		
Attitudes	0.437^**^	–	
Practice	0.162^**^	0.095^**^	-

^**^p < 0.01 (two-tailed)

### Structural equation model

**Fig. 2:**
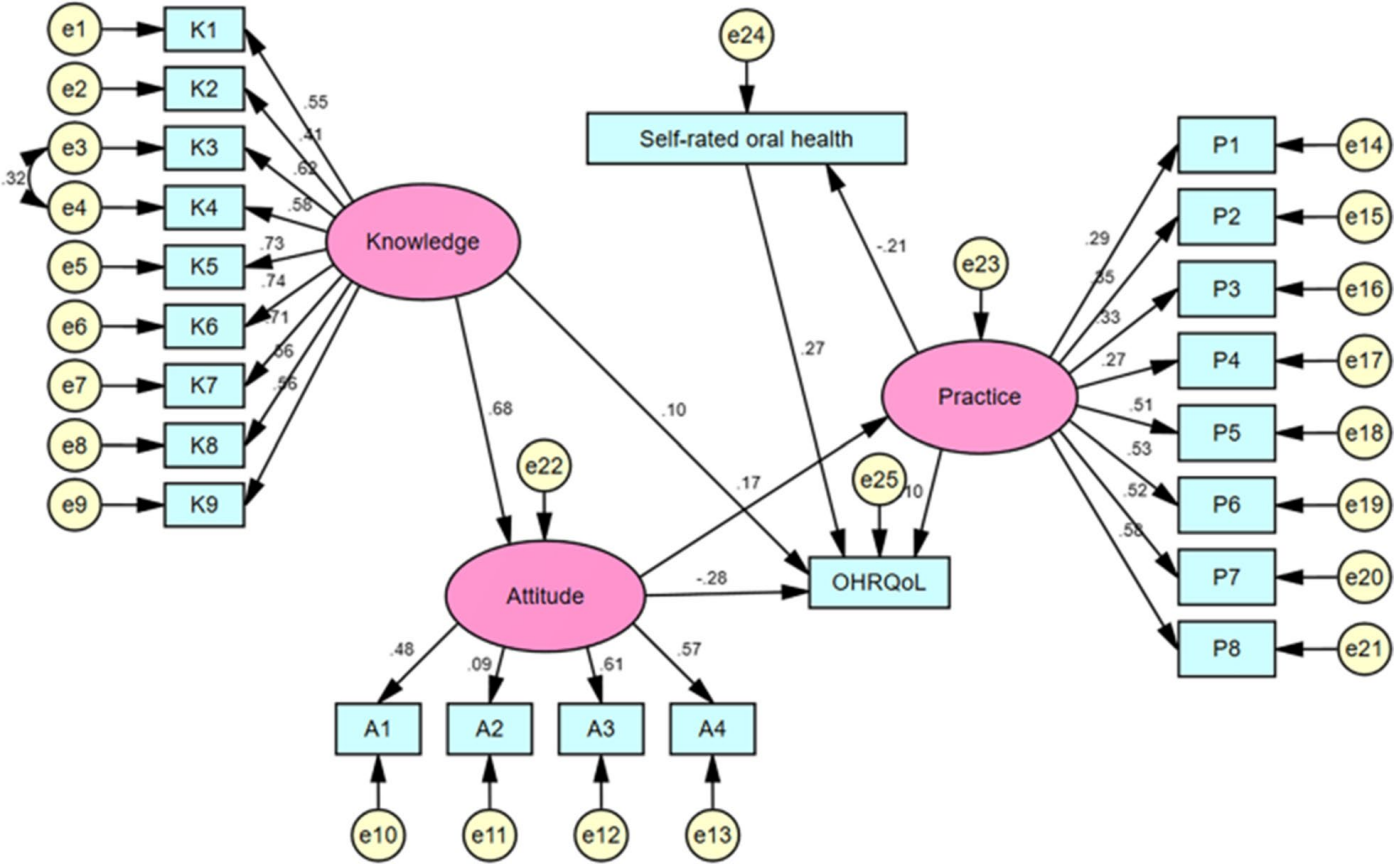
The final SEM. Rectangles show observed variables, ellipses indicate potential variables, and circles represent residual terms. The values of single-headed arrows represent the standardized coefficients. All pathways were significant (*p* < 0.05). OHRQoL was associated with oral health-related knowledge, attitudes, practice and self-rated oral health status

Figure 2 shows the final SEM model. As shown in Fig. 2 and Table 4: (1) Knowledge had a direct effect on attitudes, with a direct effect value of 0.68. The direct effect of attitudes on practice was 0.17; the indirect effect of knowledge on practice through attitudes was 0.12, 95% CI: 0.06–0.17. Therefore, attitudes play a complete mediating role between knowledge and practice. (2) Practice had a direct and negative effect on self-rated oral health with an effect value of − 0.21. (3) Students' knowledge, attitudes, practice, and self-rated oral health all had direct influences on OHRQoL (all *p* < 0.05), and the direct effect values were 0.10, − 0.28, 0.10, and 0.27, respectively.

**Table 4:** Bootstrap analysis of mediating effect significance test for the final mode

Model paths	Standardized direct effects	95% CI		Standardized indirect effects	95% CI	
		LLCI	ULCI		LLCI	ULCI
Knowledge → Attitudes	0.68***	0.60	0.75	–	–	–
Attitudes → Practice	0.17***	0.09	0.25	–	–	–
Practice → Self-rated oral health	− 0.21***	− 0.27	− 0.15	–	–	–
Self-rated oral health → OHRQoL	0.27***	0.22	0.32	–	–	–
Knowledge → Practice	–	–	–	0.12***	0.06	0.17
Knowledge → Self-rated oral health	–	–	–	− 0.03***	− 0.04	− 0.01
Attitudes → Self-rated oral health	–	–	–	− 0.04***	− 0.06	− 0.02
Knowledge → OHRQoL	0.10*	0.00	0.22	− 0.18***	− 16.63	− 6.16
Attitudes → OHRQoL	− 0.28***	− 0.40	− 0.17	0.01	− 0.10	0.85
Practice → OHRQoL	0.10 **	0.03	0.17	− 0.06***	− 8.13	− 3.40

All of the direct effects were significant (*p* < 0.05); *p < 0.05; **p < 0.01; ***p < 0.001

Among them, self-rated oral health had the greatest impact on OHRQoL (*β* = 0.27), followed by knowledge (*β* = 0.10), and practice (*β* = 0.10), and attitudes had a negative effect (*β* = − 0.28).

## Discussion

This study aimed to explore the relationships between oral health-related knowledge, attitudes, practice, self-rated oral health, and OHRQoL among Chinese college students. The results showed that oral health-related knowledge and attitudes were satisfactory, and still need to be further improved, while oral health-related practice was at a slightly lower level. Oral health knowledge directly and positively affected attitudes. Attitudes toward oral health were directly and positively associated with oral health practice. Oral health knowledge had an indirect effect on practice through attitudes. Oral health practice was directly associated with self-rated oral health. Besides, oral health knowledge, practice, and self-rated oral health were found to be directly and positively associated with OHRQoL, while attitudes were negatively associated with OHRQoL among Chinese college students. To our knowledge, this study was one of the few studies that analysed the interaction mechanism of oral health-related KAP using SEM in a group of Chinese college students. Meanwhile, the current study was the first to explore the association between oral health-related KAP, self-rated oral health, and OHRQoL in this population in China.

### Current status of oral health related KAP

To date, only a few investigations on ‘Knowledge-Attitudes-Practice’ have been conducted targeting Chinese college students' oral health based on the KAP theory. Similar studies in other countries used the Hiroshima University-Dental Behavioural Inventory (HU-DBI) questionnaire to survey dental health attitudes, perception, and behaviour [51, 52].

Our results showed that the awareness rate of oral health knowledge in Chinese college students was satisfactory. This was much higher than the results of the studies conducted by Liu et al. (37.6%) [53] and Chen, et al. (53.85%) [54] for Chinese comprehensive university students, and even higher than the findings (60.1%) from the fourth national oral health survey in the mainland of China targeting national residents [34]. This study also found that it was ideal for the holding rate of positive attitudes toward oral health, but this was lower than the findings (84.9%) of mainland residents revealed in the fourth national oral health survey of China [34]. However, our study showed that the execution rate of oral health behaviour of the participants was not optimistic, which needs improvement and deserves social attention. This result was slightly lower than the results of the study conducted by Abu-Gharbieh et al. targeting adult residents in the United Arab Emirates [55]. They reported that 53% of the participants performed better oral health behaviours. The different results of the mentioned studies may be due to the respective study design and survey instrument.

Notably, we found that the levels of oral health knowledge and attitudes were not coordinated with oral health behaviour. This might be explained by the fact that participants' acquisition of oral health knowledge did not lead to oral hygiene behavioural changes, which is a disconnect between knowledge and practice.

Spearman’s correlation analysis showed that there were positive correlations between oral health knowledge, attitudes, and practice among college students in China. This result supports the KAP theory about the causal chain of knowledge, attitudes, and practice [22]. Oral health education may be an effective strategy to improve college students’ knowledge, attitudes, and practice related to oral health [56].

The SEM model was constructed based on the KAP theory in our study. The KAP theory was developed as a human health promotion model [57]. It asserted that the change in human behaviour could be divided into three continuous processes: knowledge acquisition, belief generation, and practice/behaviour formation [57]. Knowledge, attitudes, and practice should have a positive relationship according to KAP theory [57]. In this study, the final model showed that there was a significant positive relationship between oral health knowledge and attitudes, and attitudes had a positive and direct effect on oral hygiene practice. However, oral health knowledge did not exhibit a direct relationship with practice.

Another noteworthy finding of our study was that oral health knowledge can indirectly and significantly affect oral hygiene practices through attitudes, that is, attitudes toward oral health played a mediating role in the KAP model for oral health. This finding was supported by scholars in other fields, and they also confirmed that knowledge can indirectly affect practice through attitudes [58, 59]. This indicates that obtaining oral health knowledge would motivate positive attitudes to get information about oral health and to perform oral health practice. Therefore, oral health knowledge is considered a prerequisite for oral health practice.

It is worth noting that our study demonstrated that the path coefficient for the direct effect of attitudes on practice was estimated to be *β* = 0.17, while the indirect effect of knowledge on practice was 0.12. These findings indicate that the influence of knowledge and attitudes on practice is limited. The possible reasons are explained by a limitation of the model in this study. Moreover, this may also reflect the limitation of the KAP theory in health behaviour interventions. In addition, a previous study showed that practice, apart from being correlated to both knowledge and attitudes, was also related to other factors, such as psychological factors [60, 61], level of education [62], family factors [56], and social environmental factors [63, 64].

### Oral health related KAP, self-rated oral health and OHRQoL

Our study found that oral health knowledge and practice had significant, direct, and positive effects on OHRQoL among college students, which indicated that these two variables can be effective in changing oral health-related quality of life. The results of the study conducted by Alsumait et al. [65] were similar to our research findings. They reported that oral health knowledge and practice of primary school teachers were significantly associated with their OHRQoL. Furthermore, other studies confirmed a possible association between oral hygiene practice and OHRQoL [66–68].

This study also observed that attitudes toward oral health had a negative influence on OHRQoL. This finding was inconsistent with previous research [65]. The probable reason is that turning positive attitudes into positive practice might need to take time, so the improvement in oral health-related quality of life is not obvious.

A direct negative association was found between oral health practice and self-rated oral health in this study. This demonstrates that the higher scores of oral health practice, the lower the scores of self-assessments of oral health, according to the scoring method in our questionnaire design. In other words, better oral hygiene practice can improve self-assessment of oral condition. This result is consistent with those of previous studies targeting Japanese university students [16, 69].

This study also observed that self-rated oral health status had the greatest positive effect on OHRQoL, compared with other parameters (knowledge, attitudes, and practice). This finding implies that self-rated oral health status may play a superior role in determining OHRQoL. In other words, better self-related oral health resulted in better OHRQoL. This finding was supported by a similar study conducted by Yamane et al. in Japan [16], which reported that self-rated oral health was related to OHRQoL in a young Japanese population. In addition, this association was also observed in older Australians [69].

### Analyses of deleted paths

The model and path analysis showed that the path from knowledge and attitudes to self-rated oral health did not fit the final model in this study. Furthermore, the direct path from oral health knowledge to practice was also removed. However, previous studies have shown a possible correlation between oral health knowledge, attitudes, and self-rated oral health [62, 70]. KAP theory also suggested that knowledge had a direct impact on practice [57]. The reason for the different results may be because of the respective survey design and the different demographic characteristics of participants (college students aged 22.01 ± 4.83 years from Anhui, China vs. Middle school students aged 15 to 16 years from Luma, Finland, or Lebanese pharmacists aged 39.3 ± 10.68 years) [62, 70].

### Limitations

Our study has several limitations. First, the main challenge with the use of SEM is the inability to establish inferential causality in this cross-sectional study. Second, there may be a few self-reported questionnaire design flaws in this study, which might cause potential bias and lead respondents to provide socially acceptable responses. In addition, only college students in Anhui Province (China) were included as participants in our study, which may limit the generalizability of the results. Third, in the SEM analysis, the oral health-related knowledge, attitudes, practice, self-rated oral health, and OHRQoL were included. However, other relevant variables may exist, such as individual characteristics (gender, grade, and major) and environmental characteristics (family economic annual income and parents' education level) that Wilson and Cleary had put forward [71]. The results should be interpreted with caution. Further, more large-scale in-depth studies are needed in the future.

## Conclusions

The results of this study showed that oral health knowledge and attitudes were satisfactory among Chinese college students, while their oral health practices were not optimistic. In addition, a direct positive relationship existed between oral health knowledge and attitudes. A direct positive association was found between oral health attitudes and oral health practices, and attitudes played a complete mediating role between knowledge and practice. OHRQoL was associated with oral health-related knowledge, attitudes, practice, and self-related oral health in this group of college students in China. Our findings support the KAP theory regarding the causal chain of knowledge, attitudes, and practice. Limitations of the KAP model were also found.

## Data Availability

The datasets used and/or analysed during the current study were available from the corresponding author on reasonable request.

## Abbreviations

SEM: Structural equation modelling
KAP: Knowledge, attitudes, and practices
CFI: Comparative fit index
GFI: Goodness of fit index
AGFI: Adjusted goodness of fit index
NFI: Normed fit index
RFI: Relative fit index
IFI: Incremental fit index
PNFI: Parsimonious normed fit index
PCFI: Parsimony comparative of fit index
PGFI: Parsimony goodness of fit index
TLI: Tucker-Lewis index
RMR: Root mean square residual
RMSEA: Root mean square error of approximation
MLE: Maximum likelihood estimate
SD: Standard deviation
OHRQoL: Oral health-related quality of life
CI: Confidence interval
